# Effect of Environmental Microorganisms on Fermentation Microbial Community of Sauce-Flavor *baijiu*

**DOI:** 10.3390/foods12010010

**Published:** 2022-12-20

**Authors:** Yuhan Lu, Chengnan Zhang, He Zhao, Weihong Min, Hua Zhu, Hongan Wang, Hongyun Lu, Xiuting Li, Youqiang Xu, Weiwei Li

**Affiliations:** 1 Key Laboratory of Brewing Microbiome and Enzymatic Molecular Engineering, China General Chamber of Commerce, Beijing Technology and Business University, Beijing 100048, China; 2 School of Food and Health, Beijing Technology and Business University (BTBU), Beijing 100048, China; 3 National Engineering Laboratory on Wheat and Corn Further Processing, College of Food Science and Engineering, Jilin Agricultural University, Changchun 130118, China; 4 Beijing Huadu Distillery Food Co., Ltd., Beijing 102212, China

**Keywords:** environmental microorganisms, fermented grains, Sauce-flavor *baijiu*, sourcetracker

## Abstract

The compositions of the microbial community in fermented grains of Sauce-flavor *baijiu* produced in different regions have diverse characteristics; however, the reasons for this remain unclear. The present study investigated the contributions of environmental microorganisms to the microbial community as well as the volatile compounds in the fermented grains of Sauce-flavor *baijiu* produced in the Beijing region using high-throughput sequencing combined with sourcetracker analysis, and compared the differences of environmental microorganism and their roles in the production process of Sauce-flavor *baijiu* from different regions.The results showed that the environmental microorganisms in the tools were the main contributors of the bacterial and fungal communities in fermented grains during heap fermentation and at the beginning of pit fermentation. At the end of pit fermentation, pit mud was the main environmental source of bacterial community in fermented grains, while tools and Daqu were the main environmental sources of fungal community in fermented grains.Environmental microorganisms thrived on the functional microorganisms in the fermented grains of Sauce-flavor *baijiu* produced in the Beijing region and thus shaped the profiles of volatile compounds. Environmental microorganisms of Sauce-flavor *baijiu* in the Guizhou province and the Beijing region differed significantly, which is partially responsible for the distinctive characteristics in the microbial community structure of Sauce-flavor *baijiu*-fermented grains from different regions.

## 1. Introduction

Consumers take pleasure in discussing the unique flavor characteristics of fermented foods produced in different geographical areas using similar materials and manufacturing processes. For example, the terroir is often used to describe the regionally distinctive flavor characteristics of wine [1]. Interestingly, this is also true for Sauce-flavor *baijiu*. The main regions producing Sauce-flavor *baijiu* in China include Moutai and Xishui town in the Guizhou province, Erlang town in the Sichuan province, Beijing, and Tianjin [2,3,4,5]. The flavor profiles of the Sauce-flavor *baijiu* produced in these different regions are perceived to exhibit distinctive characteristics [2,4]. Sauce-flavor *baijiu* is traditionally produced by solid-state fermentation in a semi-controlled environment, with its flavor compounds being generated mainly by the diverse and abundant microorganisms during the fermentation process [6]. Previous studies have shown that the fungal communities in the fermented grains of Sauce-flavor *baijiu* produced in the Guizhou province were dominated by *Pichia*, *Saccharomyces*, *Thermoascus*, *Wallemia*, and *Cryptococcus*, while those in Beijing were dominated by *Issatchenkia*, *Thermoascus*, and *Thermomyces* [3,4,7]. Overall, these results indicated that the composition of the microbial community in fermented grains of Sauce-flavor *baijiu* produced in different regions differed significantly and may be partially responsible for their unique flavor characteristics [6,8]. However, the reasons for the differences in the composition of these microbial communities are still unclear.

The microorganisms in the fermented grains of Sauce-flavor *baijiu* mainly originate from the Daqu starter used and the specific processing environment [7,9,10]. The traditional fermentation process for Sauce-flavor *baijiu* lasts approximately a year with seven stages, using Daqu as the starter and sorghum as the raw material [4,10]. At the end of each stage, *baijiu* is obtained by distilling the fermented sorghum (fermented grains) [4]. Each stage consists of two parts: heap fermentation and pit fermentation. During the heap fermentation process, the fermented grains are cooled to room temperature after distillation, mixed with Daqu and water, piled into cones using tools, and then fermented in an open environment for 3–5 d. During the following pit fermentation, the fermented grains are transferred to a pit and then are sprinkled evenly with tail *baijiu* (the *baijiu* obtained from the final stage of distillation). The fermented grains are then covered with rice hulls and pit mud and fermented in the pit for 30 d (Figure S1). The fermented grains thus encounter many environments, such as the tools, rice hulls, and pit mud, creating various channels for environmental microorganisms to enter and participate in the fermentation process [10,11,12]. These environmental microorganisms can compensate for any microorganism deficiencies in the Daqu used for Sauce-flavor *baijiu*, enrich the dominant functional strains, and the enhance fermentation performance [7]. Like Sauce-flavor *baijiu*, the Light-flavor and Strong-flavor *baijiu* are also manufacture in an open system [2]. The microorganisms in the processing environment of Light-flavor *baijiu* can influence the succession of the microbial community and shape metabolic profiles in the fermented grains [12]. Anaerobes in the pit mud can also migrate into the fermented grains of Strong-flavor *baijiu* and promote the generation of organic acids and flavor compounds [11]. These results have suggested that the compositions of the microbial community in fermented grains was strongly influenced by environmental microorganisms.

The environmental microorganisms in *baijiu* processing environments can differ significantly. The dominant bacterial genera in workshops where Sauce-flavor *baijiu* was produced in Guizhou province were *Sphingobacterium*, *Enterobacter*, and *Pantoea* [9], while those in workshops where Light-flavor *baijiu* was produced in the Hebei province were *Lactobacillus*, *Bacillus*, and *Weissella* [12]. However, little is known about the differences in the environmental microorganisms and their influence on the microbial community of fermented grains in the regions where Sauce-flavor *baijiu* is produced. Environmental factors, such as temperature, air pressure, and light, affect how microorganisms grow and secrete compounds into their surroundings, thereby affecting the diversity of the microbial community [9,13]. The Guizhou province has a subtropical monsoon climate, featuring hot summers and warm winters, and high humidity with little rain [14]. The Beijing and Tianjin regions, 2000 km from the Guizhou province, have a temperate monsoon climate zone with hot rainy summers and cold dry winters [15]. The present study aims to investigate the environmental microorganisms and their contribution to the composition of the microbial community of fermented grains in Sauce-flavor *baijiu* produced in the Beijing region, and to compare them with those of Sauce-flavor *baijiu* produced in the Guizhou province.

## 2. Materials and Methods

### 2.1. Sample Collection

All the samples were collected in May 2021 from a distillery in Beijing, China. The heap and pit fermentation lasted approximately 5 d and 30 d, respectively. The samples in heap fermentation were collected from the upper and lower layers when fermented for 1 d, 3 d and 5 d (Figure S1). At each location, three paralleled fermented grains samples were collected, and all these samples were grinded and mixed as fermented grains-H sample. The triplicate fermented grains samples in pit fermentation were collected from the upper and middle layers at 0 d and 30 d (Figure S1). The samples collected from pit fermentation at 0 d and 30 d were grinded and mixed as fermented grains-P0 and fermented grains-P30 samples, respectively. The triplicate sample on the tools, such as shovel and rake, were collected with sterile degreasing cotton pre-moistened with sterile 0.1 mol/L phosphate-buffered saline solution (PBS) (pH = 7). The Daqu and water used for heap fermentation and rice hulls, pit mud and tail *baijiu* used for pit fermentation were sampled in triplicate. Finally, twenty-seven samples were stored at −80 °C for subsequent analysis.

### 2.2. DNA Extraction, Bacterial 16S rRNA Gene Amplicon Sequencing, and Fungal ITS Amplicon Sequencing

The extraction, amplification, and sequencing of genomic DNA were conducted according to our previously described method [4]. In brief, microbial community genomic DNA was extracted from samples using the E.Z.N.A.^®^ soil DNA Kit (Omega Bio-tek, Norcross, GA, USA) according to manufacturer′s instructions. The V3-V4 hypervariable region of bacterial 16S rRNA gene was amplified with primer pair 338F (5′-ACTCCTACGGGAGGCAGCAG-3′) and 806R (5′-GGACTA CHVGGGTWTCTAAT-3′). The internal transcribed spacer 1 and 2 (ITS1 and ITS2) region of fungal 18S rRNA gene was amplified with the primer pair ITS1 (5′-TCCGTAGGTGAACCTGCGG-3′) and ITS2 (5′- GCTGCGTTCTTCATCGATGC-3′).

The sequencing of PCR amplification was performed on Illumina MiSeq PE300 platform/NovaSeq PE250 platform (Illumina, San Diego, CA, USA) with the protocols of Majorbio Bio-Pharm Technology Co., Ltd. (Shanghai, China). The raw bacterial 16S and fungal 18S rRNA gene sequencing reads were demultiplexed, quality filtered by fastp version 0.20.0, and merged by FLASH version 1.2.7 (Johns Hopkins University, Baltimore, MD, USA) with the following criteria: (i) the 300 bp reads were truncated at any site receiving an average quality score of <20 over a 50 bp sliding window, and the truncated reads shorter than 50 bp were discarded; reads containing ambiguous characters were also discarded; (ii) only overlapping sequences longer than 10 bp were assembled according to their overlapped sequence. The maximum mismatch ratio of overlap region is 0.2. Reads that could not be assembled were discarded. (iii) Samples were distinguished according to the barcode and primers, and the sequence direction was adjusted with exact barcode matching and 2 nucleotide mismatch in primer matching. After removing chimeric sequences, the high-quality sequences were clustered into operational taxonomic units (OTUs) with 97% similarity cutoff using UPARSE version 7.11. The taxonomic classification of each 16S rRNA and 18S rRNA gene sequences were analyzed by the RDP Classifier version 2.2 (Michigan State University, East Lansing, MI, USA) against the SILVA v138 rRNA and greengene database at a confidence level of 70%, respectively [4].

### 2.3. Volatile Compounds Analysis

The volatile compounds were quantified according to our previously described method [4]. Briefly, the samples were mixed with saturated NaCL solution and 4-octanol as an internal standard and then ultrasonically treated for 30 min. The volatile compounds were extracted using a 50/30 μm DVB/CAR/PDMS fiber (Supelco, Bellefonte, PA, USA) and analyzed using HS-SPME-GC-MS (TSQ 8000 Evo, Trace MS/GC, Thermo Fisher Scientific, Waltham, MA, USA) equipped with a TG- 5MS column (30 m × 0.25 mm × 0.25 μm, J&W Scientific, Folsom, CA, USA) and a flame ionization detector. The GC-MS conditions were: a starting temperature of 40 °C holding for 3 min, which was then increased to 100 °C at a rate of 2 °C/min and held at 100 °C for 5 min, and then increased to 150 °C with a rate of 2 °C/min and held at 150 °C for 2 min, and finally increased to 230 °C with a rate of 10 °C/min and held at 230 °C for 5 min [4]. Mass spectra was generated in the electron ionization mode at 70 eV ionization energy. The full scan mode, which ranged from 28 to 500 amu, was employed [4]. The quantitative analysis of volatile substances was performed by matching mass spectra with the NIST05 spectral database.

### 2.4. Data Analysis

All the assays were conducted in triplicate and the results were presented as mean with standard deviations. Principle coordinate analysis (PCoA) was employed to evaluate the ecological distances of different samples on a genus level based on unweighted UniFrac distances. Sourcetracker (v0.9.8) (University of Colorado, Boulder, CO, USA) was used to analyze the source of the microbial communities in fermented grains. Fermented grains-H, fermented grains-P0, and fermented grains-P30 were set as sink, and rice hulls, tools, pit mud, water, tail *baijiu*, and Daqu were set as source. The Spearman rank correlations between volatile compounds and the relative abundance of twenty-three shared genera in fermented grains and environments were calculated using OriginPro2019 (OriginLab Corporation, MA, USA) and visualized using a heatmap diagram. The statistically significant differences in Shannon and Chao 1 indexes were analyzed using one-way analysis of variance (ANOVA) in OriginPro2019 (OriginLab Corporation, MA, USA) (*p* ≤ 0.05, Duncan’s test).

## 3. Results

### 3.1. Bacterial Community Compositions of Daqu, Fermented Grains, and Environmental Samples

**Figure 1:**
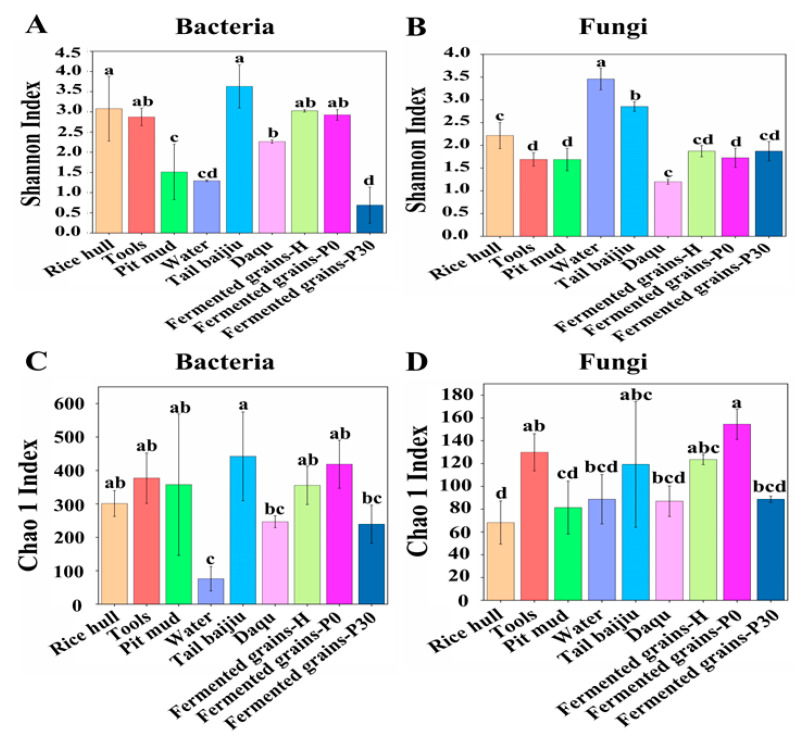
Alpha diversity of microbial community in Daqu, environment, and fermented grains during the heap and pit fermentation of Sauce-flavor *Baijiu*. Shannon index values of bacterial (**A**) and fungal communities (**B**). Chao 1 index values of bacterial (**C**) and fungal communities (**D**). a, b, c, d means in columns labeled with different letters differed significantly at *p* < 0.05.

The compositions of the bacterial community in Daqu, fermented grains, and environmental samples were analyzed via high-throughput sequencing. A total of 2,358,795 high-quality reads from the V3-V4 regions of the bacterial rDNA gene in 27 samples were obtained, with a range from 78,011 to 115,346 reads. The rarefaction curves based on the OTU number gradually flattened, indicating that the sequencing depth was sufficient to capture the vast majority of phylotypes in the samples (Figure S2). Alpha diversity expressed as Shannon and Chao1 indexes reflected the degree of the microbial community diversity and richness [1]. The bacterial community diversity of tail *baijiu*, rice hulls, tools and Daqu were significantly higher than those of pit mud and water (Figure 1A).The bacterial community richness and diversity in fermented grains at the end of pit fermentation was lowest among all samples (Figure 1A,C).

**Figure 2:**
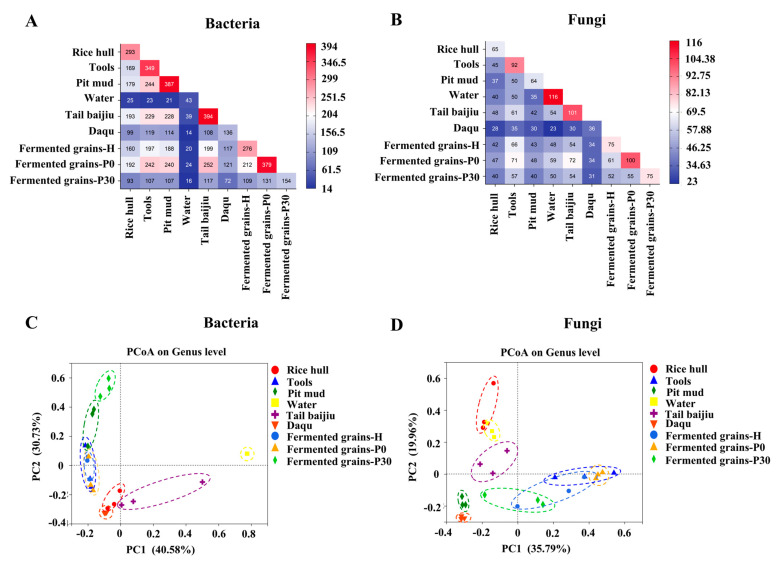
Sharing of microbial taxa among samples: (**A**): Shared bacterial taxa; (**B**): Shared fungal taxa and Beta diversity assessed by using unweighted UniFrac principle coordinate analysis (PCoA) plot based on genus level ((**C**): Bacteria; (**D**): Fungi).

**Figure 3:**
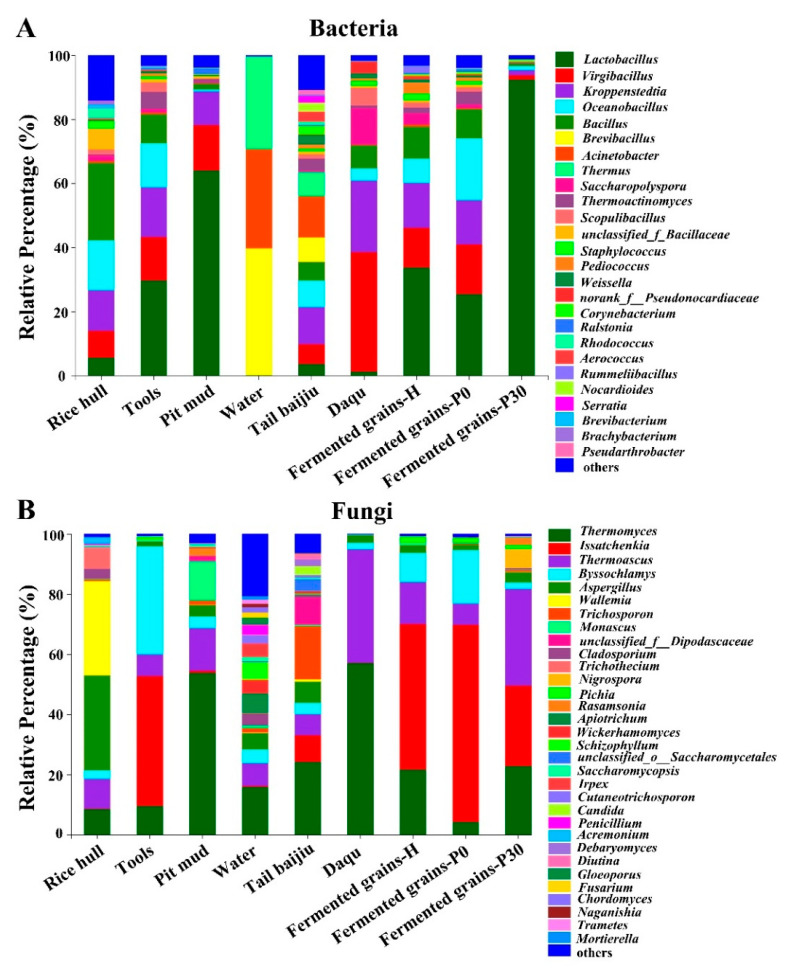
Microbial community structure in Daqu, environment, and fermented grains during the heap and pit fermentation of Sauce-flavor *baijiu*. Profile of bacterial taxa at the genus level (**A**). Profile of fungal taxa at the genus level (**B**).

The taxonomy of bacterial OTU sequences were further annotated based on SILVA rRNA and greengene database. A total of 750 bacterial genera were identified in all samples. The shared genera among Daqu, fermented grains, and environmental samples were investigated (Figure 2A). Altogether, 276 genera were detected in fermented grains during heap fermentation, of which 199 genera could be detected in tail *baijiu*, 197 genera in tools, 188 genera in pit mud, 160 genera in rice hull, and 117 genera in Daqu. At the beginning of pit fermentation, the number of genera in fermented grains increased to 379, with a 126.63%, 122.84%, 127.66%, and 120% increase in genera shared with tail *baijiu*, tools, pit mud, and rice hull, respectively. At the end of pit fermentation, shared bacterial genera in fermentation grains, Daqu, and environmental samples decreased significantly (Figure 3A). For the environmental samples, pit mud and tools, as well as tail *baijiu*, tools, and pit mud, had high numbers of shared genera (Figure 2A).

For Daqu and environmental samples, *Virgibacillus* and *Kroppenstedtia* shared predominant genera (average relative abundance greater than 10%) in Daqu, tools, and pit mud samples, while *Saccharopolyspora*, *Scopulibacillus*, and *unclassified_f_Bacillaceae* shared subdominant genera (average relative abundance between 10% and 1%) in rice hulls and tools (Figure 3A). *Lactobacillus* was predominant in tools and pit mud while subdominant in Daqu, rice hulls, and tail *baijiu* (Figure 3A). *Bacillus* was more abundant in rice hulls but less abundant in Daqu, tools, pit mud, and tail *baijiu*. For fermented grain samples, *Lactobacillus*, *Virgibacillus*, and *Kroppenstedtia* shared predominant genera and *Saccharopolyspora*, *Thermoactinomyces*, *Scopulibacillus*, *Staphylococcus*, and *Pediococcus* shared subdominant genera in fermented grains-H and fermented grains-P0 (Figure 3A). *Lactobacillus* was dominant in the fermented grains-P30, accounting for 84.12–97.25%.

Principal coordinate analysis (PCoA) was further carried out based on the profile of the bacterial community (Figure 2C). The two axes explained 71.31% of differences in the compositions of the bacterial community. Results showed that fermented grains-H and fermented grains-P0 were similar with tools, while pit mud was between fermented grains-P0 and fermented grains-P30 (Figure 2C).

### 3.2. Environmental Contribution to Bacterial Community in Fermented Grains

**Figure 4:**
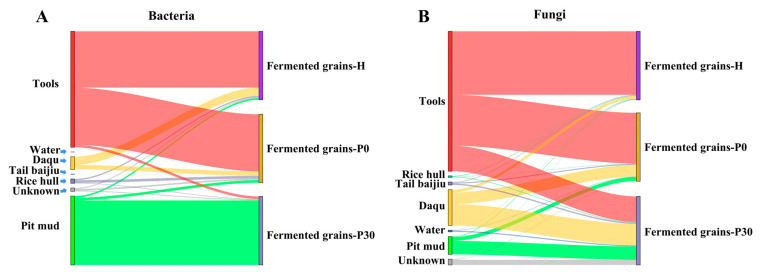
Microbial source estimation by Sourcetracker: (**A**) Source-tracking analysis of bacterial communities in fermented grains during the heap and pit fermentation of Sauce-flavor *baijiu*. (**B**) Source-tracking analysis of fungal communities in fermented grains during the heap and pit fermentation of Sauce-flavor *baijiu*.

**Figure 5:**
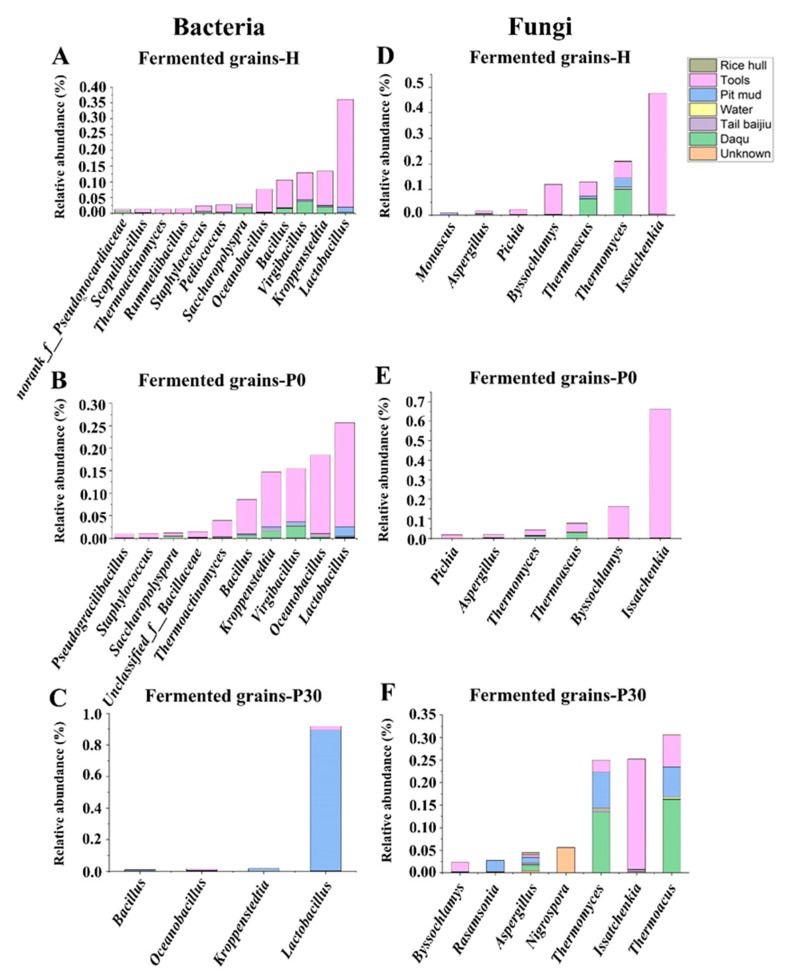
Source-tracking analysis of bacterial genus in fermented grains during the heap fermentation (**A**), beginning (**B**), and end (**C**) of pit fermentation. Source-tracking analysis of fungal genus in fermented grains during the heap fermentation (**D**), beginning (**E**), and end (**F**) of pit fermentation.

The Sourcetracker was applied to track the sources of bacteria in fermented grains. The results showed that tools was the main contributor of the bacterial communities in fermented grains-H and fermented grains-P0 (Figure 4A). Tools contributed 34.13% *Lactobacillus*, 10.89% *Kroppenstedtia*, 8.62% *Virgibacillus*, 8.79% *Bacillus*, 7.27% *Oceanobacillus*, 2.30% *Pediococcus*, and 1.80% *Staphylococcus* in fermented grains during heap fermentation (Figure 5A), and contributed 23.13% *Lactobacillus*, 17.42% *Oceanobacillus*, 11.88% *Virgibacillus*, 12.13% *Kroppenstedtia*, 7.48% *Bacillus*, and 3.55% *Thermoactinomyces* in fermented grains at the beginning of pit fermentation (Figure 5B). Pit mud was the main environmental source of the bacterial community in fermented grains-P30, contributing 89.30% *Lactobacillus* in fermented grains at the end of pit fermentation (Figure 5C).

### 3.3. Fungal Community Compositions of Daqu, Fermented Grains, and Environmental Samples

A total of 3,111,514 high-quality reads from internal transcribed spacer (ITS) regions of the fungal rDNA gene in 27 samples were obtained, with a range from 106,734 to 124,340 reads. For the Daqu and environmental samples, the fungal community diversity of tail *baijiu* and water were significantly higher than that of rice hulls, tools, pit mud, and Daqu (Figure 1B). The fungal community richness of tools and tail *baijiu* were remarkably higher than that of rice hulls, pit mud, water, and Daqu (Figure 1D). For fermented grain samples, we found that the differences in the fungal community diversity among samples were not significant (Figure 1B); however, fermented grains at the beginning of pit fermentation had the highest fungal community richness (Figure 1D).

The fungal taxa at genus level in samples were analyzed (Figure 3B). A total of 212 fungal genera were identified in all samples. For fermented grains, 75 genera were detected in samples during heap fermentation, of which 66 genera were shared with tools, 54 genera were shared with tail *baijiu*, 48 genera were shared with water, 43 genera were shared with pit mud, and 42 genera were shared with rice hulls (Figure 2B). At the beginning of pit fermentation, genera in fermented grains increased to 100, with a 133.33%, 122.91%, 111.62%, 107.58%, and 111.90% increase in genera shared with tail *baijiu*, water, pit mud, tools, and rice hulls, respectively. At the end of pit fermentation, the profile of shared fungal genera between fermentation grains, Daqu, and environmental samples was similar to that during heap fermentation (Figure 2B). For environmental samples, tools and tail *baijiu*, and water as well as pit mud, had relatively higher numbers of shared genera (Figure 2B).

For Daqu and environmental samples, *Thermomyces* shared predominant genera in Daqu, tail *baijiu*, water, and pit mud, and *Thermoascus* shared subdominant genera in rice hulls, tools, water, and tail *baijiu* (Figure 3B). *Byssochlamys* was predominant in tools while subdominant in other environmental samples. *Aspergillus* was predominant in rice hulls while subdominant in other environmental samples. *Issatchenkia* was more abundant in tools but less abundant in tail *baijiu* (Figure 3B). For fermented grain samples, *Issatchenkia* shared predominant genera and *Aspergillus*, and *Pichia* shared subdominant genera among fermented grains samples (Figure 3B). *Thermomyces* and *Thermoascus* shared predominant genera in fermented grains-H and fermented grains-P30, while there were subdominant genera in fermented grains-P0. *Byssochlamys* was more abundant in the fermented grains-P0 while less abundant in other fermented grain samples (Figure 3B).

PCoA was further performed based on the profile of the fungal community (Figure 2D). The results showed that fermented grains-H was between fermented grains-P0 and fermented grains-P30. Daqu, pit mud, and tail *baijiu* were closed to fermented grains-P30 (Figure 2D).

### 3.4. Environmental Contribution to Fungal Community in Fermented Grains

Sourcetracker analysis showed that tools was the major contributor of the fungal communities in fermented grains-H and fermented grains-P0 and a partial contributor to the fungal community in fermented grains-P30 (Figure 4B). Tools contributed 47.20% *Issatchenkia*, 11.60% *Byssochlamys*, 6.40% *Thermomyces*, 5.50% *Thermoascus*, and 2.00% *Pichia* in fermented grains during heap fermentation (Figure 5D), and contributed 70.00% *Issatchenkia* and 20.00% *Byssochlamys* in fermented grains at the beginning of pit fermentation (Figure 5E). At the end of pit fermentation, 24.00% *Issatchenkia*, 7.00% *Thermoascus*, 2.00% *Thermomyces*, 2.00% *Byssochlamys*, and 1.00% *Aspergillus* were derived from tools (Figure 5F).

Daqu was another main contributor to the fungal communities in fermented grains-P0 and fermented grains-P30 (Figure 4B). Daqu contributed 2.95% *Thermoascus* and 1.02% *Thermomyces* at the beginning of pit fermentation, while contributed 16.19% *Thermoascus* and 13.53% *Thermomyces* at the end of pit fermentation (Figure 5E,F). In addition, 8.08% *Thermomyces*, 6.63% *Thermoascus*, 2.47% *Rasamsonia*, and 1.26% *Aspergillus* in the fungal community of fermented grains-P30 were originated from pit mud (Figure 5F).

### 3.5. Correlation between Volatile Compounds and Microbes from Daqu and Environment in Fermented Grains

To explore the contributions of microorganisms in fermented grains from Daqu and environment to the flavor of *baijiu*, the volatile compounds of Daqu and fermented grains were analyzed. A total of 56 volatile compounds were detected in Daqu and fermented grain samples, including 5 acids, 7 alcohols, 3 aldehydes, and 28 esters (Tables S1 and S2). The variety and concentrations of esters in fermented grains at the end of pit fermentation were the highest among samples (Table S1).

The Spearman correlation coefficients between relative abundance of dominant microorganisms from Daqu and the environment in fermented grains and concentrations of key volatiles were further calculated. Results showed that these microorganisms were important contributors to the formation of volatile compounds (Table S2). Among them, *Issatchenkia*, as well as *Oceanobacillus*, were closely related to nine esters, including ethyl 9-hexadecenoate, ethyl iso-allocholate and ethyl acetate.. *Byssochlamys* exhibited positive correlation with nine esters and hexadecanoic acid. *Lactobacillus* was closely related to four acids, including acetic acid, isobutyric acid, butyric acid, hexanoic acid, and 18 esters including ethyl caproate, ethyl lactate, ethyl succinate, ethyl dodecanoate (Table S2).

## 4. Discussion

This study investigated the contribution of microorganisms originating from Daqu and the environment to the microbial community and flavor compounds in the fermented grains of Sauce-flavor *baijiu* produced in the Beijing region. During the heap fermentation of Sauce-flavor *baijiu*, the fermented grains are first mixed thoroughly with Daqu and water, and then placed in an open environment for fermentation [16,17,18]. A total of 123 bacterial and 59 fungal genera were common to the fermented grains with Daqu and water, accounting for 44.57% and 78.67% of the total number of bacterial and fungal genera in the fermented grains, respectively (Figure 2A,B). The microorganisms in the fermented grains and the environment are forced to migrate, both artificially and naturally, thus enriching the diversity of the microbial community and promoting the generation of flavor compounds [7,11,13]. Sourcetracker analysis showed that the microorganisms on the tools contributed significantly to the microbial community of fermented grains during heap fermentation (Figure 4A,B), indicating that environmental microorganisms enhance its richness [19]. Similarly, the number of bacterial and fungal genera in the fermented grains was found to increase significantly at the beginning of pit fermentation compared to the situation in the heap fermentation (Figure 1C,D and Figure 2A,B), with an increase in the number of microorganisms originating from the tools, rice hulls, and pit mud (Figure 4A,B). These results indicated that environmental microorganisms are an important source of microbes in the fermented grains of Sauce-flavor *baijiu* produced in the Beijing region.

The environmental microbiota thrived with the functional microorganisms in the fermented grains of Sauce-flavor *baijiu* produced in the Beijing region, thereby shaping the profiles of those compounds related to flavor. An analysis of the microbial taxonomic profile showed that the relative abundance of *Lactobacillus*, *Oceanobacillus*, *Issatchenkia*, and *Byssochlamys* was low in Daqu but high in the processing environment (Figure 3A,B). These genera, mainly derived from the tools, were dominant during the heap fermentation and at the beginning of pit fermentation (Figure 3A,B and Figure 5A,B), indicating that environmental microorganisms had fueled the increase in the relative abundance of these genera in the fermented grains. *Lactobacillus*, which is abundant in the fermented grains of Light-flavor, Strong-flavor, and Sauce-flavor *baijiu*, is considered to be the main functional genus during the fermentation process of *baijiu* [11,20]. *Lactobacillus* can synthesize lactic acid, acetic acid, ethyl lactate, ethyl acetate, and other compounds related to flavor by producing esterase and lipase, thus providing the fruit flavor and mellow feeling of *baijiu* [11,20]. *Oceanobacillus* can produce enzymes, such as amylase, lipase, and esterase, as well as lactic acid [20,21,22]. *Byssochlamys* can actively participate in the saccharification and fermentation processes by producing pectinase and lipase [16], which are closely related to the synthesis of ethyl hexanoate and ethyl octanoate [20]. *Issatchenkia*, which is abundant in the fermented grains of Sauce-flavor and Miscellaneous-flavor *baijiu* as a non-Saccharomyces fungal genus, can produce β-glycosidase, as well as phenethyl alcohol, various acids, and ethyl esters [23,24,25,26,27]. In the present study, the Spearman rank correlation analysis showed that the concentrations of several acids, such as hexanoic acid, butyric acid, isobutyric acid, and acetic acid, as well as ethyl esters such as ethyl acetate, ethyl lactate, ethyl caproate, and ethyl dodecanoate, were positively correlated with the relative abundance of *Lactobacillus*, *Oceanobacillus*, *Issatchenkia*, and *Byssochlamys* (Table S2). This indicated that these microorganisms, which mainly originate from the environment and dominate in the fermented grains, played an important role in the production of flavor compounds.

The environmental microorganisms in the manufacturing environments of Sauce-flavor *baijiu* differed significantly and were partially responsible for differences in the structure of the microbial community in the fermented grains of Sauce-flavor *baijiu* produced in different regions. The Guizhou province is the main area producing Sauce-flavor *baijiu* [9]. The results on the compositions of the fungal community on the tools used during the production of Sauce-flavor *baijiu* in the Guizhou province showed that the relative abundance of *Pichia*, *Torulaspora*, and *Wickerhamomyces* were high and dominant [7], while, in the Beijing region, the relative abundance of *Thermomyces*, *Issatchenkia*, *Thermoascus*, and *Byssochlamys* on tools were high and dominant (Figure 3). This difference in environmental microorganisms from different regions can not only be attributed to the differences in environmental conditions, but also to the influence of long-standing activity in brewing Sauce-flavor *baijiu*. As the brewing of batches of Sauce-flavor *baijiu* is repeated, the dominant microorganisms in the fermented grains with a strong environmental tolerance gradually establish a stable microbial community in that particular environment [12]. These environmental microorganisms interact with those in the fermented grains, thus profoundly influencing the composition and metabolism of the microbial community [7,11,12]. The present study compared the composition of the microbial community in fermented grains of Sauce-flavor *baijiu* produced in Guizhou province and the Beijing region, and found that *Lactobacillus*, *Virgibacillus*, *Kroppenstedtia*, *Oceanobacillus*, *Bacillus*, and *Pediococcus* were the dominant shared bacterial genera [4,7,16,17,18,20], with *Pichia*, *Saccharomyces*, *Thermoascus*, *Aspergillus*, *Byssochlamys*, and *Monascus* being the dominant shared fungal genera [4,7,10,16,17]; however, their relative abundance differed significantly. Specifically, *Pichia* was more abundant in fermented grains from Guizhou province, while *Issatchenkia* was more plentiful in fermented grains from the Beijing region. The succession of the microbial community in the fermented grains was strongly influenced by various environmental conditions such as the temperature, oxygen level, and water activity [6,9]. Differences in the environmental conditions between the Beijing region and the Guizhou province could partially account for differences in the relative abundance of the shared dominant microbial genera. *Pichia*, the core non-Saccharomyces fungal genus in the fermented grains of Guizhou province, can produce toxins and organic acids which inhibit the growth of filamentous fungi and bacillus, thus influencing the succession of the microbial community. *Pichia* can also shape the profiles of flavor compounds in the fermented grains by producing several alcohols, acids, and esters [7]. A previous study has shown that *Pichia* in the fermented grains of Guizhou province mainly originated from tools and the indoor environment rather than from the Daqu [7]. Similarly, *Issatchenkia* in fermented grains of *baijiu* from the Beijing region also originated from the tools rather than from the Daqu (Figure 5A,B). Overall, these results have indicated that differences in the composition of microorganisms distributed in the environments where Sauce-flavor *baijiu* is produced contributed to differences in the fermentation microbiota.

## 5. Conclusions

The present study investigated the contributions of environmental microorganisms to the microbial communities and flavor components in fermented grains of Sauce-flavor *baijiu* from the Beijing region. The results showed that tools were the main contributors of the bacterial and fungal communities in fermented grains during heap fermentation and at the beginning of pit fermentation. Tools contributed 47.20% *Issatchenkia*, 34.13% *Lactobacillus*, 11.60% *Byssochlamys*, 10.89% *Kroppenstedtia*, 8.62% *Virgibacillus*, 8.79% *Bacillus*, 7.27% *Oceanobacillus*, 6.40% *Thermomyces*, 5.50% *Thermoascus*, 2.30% *Pediococcus*, 2.00% *Pichia*, and 1.80% *Staphylococcus* in fermented grains during heap fermentation, and contributed 70.00% *Issatchenkia*, 23.13% *Lactobacillus*, 20.00% *Byssochlamys*, 17.42% *Oceanobacillus*, 12.13% *Kroppenstedtia*, 11.88% *Virgibacillus*, 7.48% *Bacillus*, and 3.55% *Thermoactinomyces* in fermented grains at the beginning of pit fermentation. Pit mud contributed 89.30% *Lactobacillus*, 8.08% *Thermomyces*, 6.63% *Thermoascus*, 2.47% *Rasamsonia*, and 1.26% *Aspergillus* in fermented grains at the end of pit fermentation, which was the main environmental source of the bacterial community in fermented grains at the end of pit fermentation. Tools and Daqu were the main contributors to the fungal communities in fermented grains at the end of pit fermentation. At the end of pit fermentation, 24.00% *Issatchenkia*, 7.00% *Thermoascus*, 2.00% *Thermomyces*, 2.00% *Byssochlamys*, 1.00% *Aspergillus* were came from tools, while 16.19% *Thermoascus*, and 13.53% *Thermomyces* were originated from Daqu. The environmental microorganisms on the tools, pit mud, and Daqu shaped the profile of the volatile compounds in the fermented grains. The environmental microorganisms of Sauce-flavor *baijiu* from Guizhou province and the Beijing region differed significantly, which partially contributed to the distinctive characteristics in the compositions of the microbial community structure in fermented grains.

## Data Availability

Data are contained within the article or the Supplementary Materials.

## Supplementary Materials

The following supporting information can be downloaded at: https://www.mdpi.com/article/10.3390/foods12010010/s1, Figure S1: Schema of Sauce-flavor Baijiu production (A). Sampling positions during heap fermentation (B) and pit fermentation (C). Sampling and experimental processes (D); Figure S2: Rarefaction curves of Shannon index (A,C) and Chao1 index (B,D) for different samples; Table S1: Analysis of volatile compounds in Daqu and fermented grains during the heap and pit fermentation of Sauce-flavor Baijiu (mg/kg); Table S2: Spearman’s correlation coefficients between shared microorganisms and volatile flavors compounds.
