# Supplementary material for: Effect of Environmental Microorganisms on Fermentation Microbial Community of Sauce-Flavor baijiu
Source: Foods. 2022 Dec 20;12(1):10. doi: 10.3390/foods12010010 (PMC9818559; doi:10.3390/foods12010010)
Supplement: Supplementary file 1 [file foods-12-00010-s001.zip › foods-2053838-supplementary.pdf]

Supplementary Materials

Effect of Environmental Microorganisms on Fermentation Microbial Community of Sauce-Flavor *baijiu*

Yuhan Lu [1,2,†], Chengnan Zhang [1,2,†], He Zhao [1,3], Weihong Min [3], Hua Zhu [4], Hongan Wang [4], Hongyun Lu [1,2], Xiuting Li [1,2,*], Youqiang Xu [1,2] and Weiwei Li [1,2]

[1] Key Laboratory of Brewing Microbiome and Enzymatic Molecular Engineering, China General Chamber of Commerce, Beijing Technology and Business University, Beijing 100048, China
[2] School of Food and Health, Beijing Technology and Business University (BTBU), Beijing 100048, China
[3] National Engineering Laboratory on Wheat and Corn Further Processing, College of Food Science and
Engineering, Jilin Agricultural University, Changchun 130118, China
[4] Beijing Huadu Distillery Food Co., Ltd., Beijing 102212, China
* Correspondence: lixt@btbu.edu.cn
† These authors contribute equally to this work.

Supplementary Table

Table S1. Analysis of volatile compounds in Daqu and fermented grains during the heap and pit fermentation of Sauce-flavor Baijiu (mg/kg).

Volatile compounds	Daqu	Fermented grains-H	Fermented grains-P0	Fermented grains-P30
Hexadecanoic acid	ND	0.097±0.040	0.061±0.030	0.062±0.020
Acetic acid	ND	ND	ND	0.932±0.520
Isobutyric acid	ND	ND	ND	0.172±0.020
Butyric acid	ND	ND	ND	0.637±0.110
Hexanoic acid	ND	0.251±0.060	0.290±0.050	0.521±0.440
1-Pentanol	ND	ND	0.068±0.010	ND
Phenylethyl Alcohol	ND	2.887±0.790	2.661±0.080	2.808±0.790
Ethanol	0.855±0.68	ND	ND	0.594±0.520
1-Heptanol	ND	0.044±0.020	ND	ND
Nicotinyl alcohol	ND	0.416±0.100	ND	ND
3-Furanmethanol	ND	0.485±0.040	0.410±0.110	ND
Undecaethylene glycol	ND	0.005±0.000	ND	ND
Benzaldehyde	ND	0.056±0.040	ND	0.073±0.020
5-Hydroxymethylfurfural	ND	0.011±0.000	ND	ND
Furfural	ND	0.996±0.150	ND	ND
Ethyl iso-allocholate	ND	ND	0.005±0.000	ND
Ethyl Acetate	ND	0.013±0.000	0.013±0.000	0.006±0.000

Ethyl caproate	ND	0.057±0.040	0.160±0.100	0.595±0.440
Ethyl lactate	ND	0.782±0.170	0.821±0.090	1.187±0.300
Ethyl 9-hexadecenoate	ND	0.004±0.000	0.007±0.000	ND
Cyclopropanetetradecanoic acid, 2-octyl-,methyl ester	0.189±0.24	ND	ND	0.164±0.010
Methyl anthranilate	0.031±0.03	ND	ND	ND
[1,1'-Bicyclopropyl]-2-octanoic acid, 2'-hexyl-, methyl ester	ND	ND	0.009±0.000	ND
9-Octadecenoic acid, (2-phenyl-1,3-dioxolan-4-yl)methyl ester,trans	ND	ND	0.025±0.000	ND
6,9,12,15-Docosatetraenoic acid, methyl ester	0.028±0.020	ND	ND	ND
Ethyl benzeneacetate	ND	0.117±0.070	0.115±0.050	0.201±0.140
β-Phenethyl acetate	ND	0.194±0.080	0.117±0.060	0.126±0.100
Ethyl dodecanoate	ND	0.062±0.010	0.098±0.060	0.127±0.060
Ethyl tetradecanoate	ND	0.331±0.070	0.308±0.010	0.384±0.140
Ethyl hexadecanoate	ND	3.466±0.990	2.789±2.410	0.959±1.650
DL-Phenylalanine,N-chlorodifluoroacetyl-,ethyl ester	ND	0.099±0.030	0.061±0.020	ND
9-Octadecenoic acid,(2-phenyl-1,3-dioxolan-4-yl)methyl ester,cis	ND	0.008±0.000	0.008±0.000	0.010±0.000
9-Octadecenoic acid,(2-	ND	0.036±0.010	ND	0.046±0.010

phenyl-1,3-dioxolan-4-yl)methyl ester,trans				
Ethyl succinate	ND	0.252±0.050	0.242±0.010	0.393±0.030
Ethyl (9E)-9-octadecenoate	ND	1.103±0.200	0.985±0.060	1.395±0.660
Ethyl 9,12,15-octadecatrienoate	ND	0.063±0.010	0.060±0.000	0.076±0.030
9,12,15-Octadecatrienoic acid,2,3-dihydroxypropyl ester, (Z,Z,Z)-	ND	ND	ND	0.031±0.010
Ethyl-9,12-octadecadienoate	0.020±0.020	0.042±0.030	0.034±0.030	0.062±0.030
Dibutyl phthalate	0.046±0.040	ND	ND	0.032±0.010
Phthalic acid, di(2-propylpentyl) ester	ND	0.008±0.000	0.024±0.010	0.042±0.010
[1,1'-Bicyclopropyl]-2-octanoic acid,2'-hexyl-, methyl ester	ND	ND	0.012±0.010	ND
Ethyl 13-methyl-tetradecanoate	ND	ND	0.068±0.000	0.078±0.040
n-Propyl 9,12-octadecadienoate	ND	2.318±0.440	2.163±0.220	2.634±1.140
Butyl 9,12-octadecadienoate	0.019±0.020	ND	ND	0.040±0.020
Ethyl 9.cis.,11.trans.-octadecadienoate	ND	0.026±0.010	0.028±0.020	ND
Nitrosomethane	ND	ND	ND	0.636±0.470
1,2-Dimethoxybenzene	ND	ND	ND	0.972±1.330
Benzeneethanamine, 3-fluoro-á,5-dihydroxy-N-	ND	ND	0.034±0.000	ND

methyl				
(2-Aziridinylethyl)amine	ND	0.025±0.010	0.024±0.010	ND
Phenol	0.076±0.030	ND	0.037±0.010	ND
2,7-Diphenyl-1,6-dioxopyridazino[4,5:2',3']pyrrolo[4',5'-d]pyridazine	0.004±0.000	ND	ND	ND
(2S,2'S)-2,2'-Bis[1,4,7,10,13-pentaoxacycl opentadecane]	0.190±0.150	ND	ND	0.031±0.010
4-Methoxy-3-methylphenol	ND	ND	ND	0.012±0.000
Heptaethylene glycol monododecyl ether	ND	ND	0.022±0.020	0.007±0.000
Tetraacetyl-d-xylonic nitrile	ND	ND	0.010±0.000	ND
2-Acetylpyrrole	ND	0.054±0.020	0.042±0.000	0.044±0.010

ND: not detected.

Table S2 Spearman's correlation coefficients between shared microorganisms and volatile flavors compounds.

Microorganisms	*Bacillus*		*Kroppenstedtia*		*Lactobacillus*		*norank_f__ Pseudonocardiaceae*		*Oceanobacillus*		*Pediococcus*		*Rummeliibacillus*		*Saccharopolyspora*		*Scopulibacillus*	
Volatile compounds	*r*	*p*	*r*	*p*	*r*	*p*	*r*	*p*	*r*	*p*	*r*	*p*	*r*	*p*	*r*	*p*	*r*	*p*
Hexadecanoic acid	0.41	0.27	0.59	0.09	-0.39	0.29	0.41	0.28	0.14	0.73	0.32	0.39	0.03	0.95	0.27	0.48	**0.75**	**0.02**
Acetic acid	**-0.81**	**0.01**	**-0.69**	**0.04**	**0.79**	**0.01**	-0.43	0.25	-0.57	0.11	-0.39	0.31	-0.22	0.56	-0.61	0.08	-0.39	0.30
Isobutyric acid	**-0.96**	**0.00**	**-0.84**	**0.00**	**0.95**	**0.00**	-0.49	0.19	**-0.68**	**0.04**	-0.44	0.24	-0.25	0.51	**-0.70**	**0.04**	-0.44	0.23
Butyric acid	**-0.95**	**0.00**	**-0.83**	**0.01**	**0.95**	**0.00**	-0.48	0.19	**-0.68**	**0.05**	-0.43	0.24	-0.25	0.51	**-0.69**	**0.04**	-0.44	0.24
Hexanoic acid	-0.44	0.23	-0.30	0.44	0.36	0.34	-0.30	0.43	-0.21	0.60	-0.24	0.53	-0.26	0.50	-0.38	0.32	-0.22	0.56
1-Pentanol	0.39	0.30	0.40	0.28	-0.55	0.13	-0.48	0.19	**0.77**	**0.01**	-0.44	0.24	-0.25	0.51	-0.15	0.70	-0.44	0.24
Phenylethyl Alcohol	0.03	0.93	0.19	0.62	-0.08	0.84	0.07	0.86	-0.07	0.87	0.21	0.58	-0.51	0.16	0.10	0.81	0.38	0.32
Ethanol	**-0.74**	**0.02**	-0.66	0.05	**0.74**	**0.02**	-0.37	0.33	-0.54	0.14	-0.33	0.38	-0.19	0.62	-0.53	0.14	-0.33	0.38
1-Heptanol	0.58	0.10	0.57	0.11	-0.41	0.27	**0.86**	**0.00**	-0.13	0.74	**0.77**	**0.01**	0.31	0.42	**0.73**	**0.03**	**0.99**	**0.00**

Nicotinyl alcohol	0.53	0.14	0.46	0.22	-0.40	0.29	**0.94**	**0.00**	-0.13	0.74	**0.92**	**0.00**	0.29	0.44	**0.87**	**0.00**	**0.93**	**0.00**
3-Furanmethanol	-0.42	0.26	-0.33	0.39	0.25	0.52	**-0.94**	**0.00**	0.28	0.46	**-0.85**	**0.00**	-0.49	0.18	**-0.78**	**0.01**	**-0.85**	**0.00**
Undecaethylene glycol	0.54	0.13	0.43	0.25	-0.40	0.29	**0.98**	**0.00**	-0.13	0.73	**0.92**	**0.00**	0.43	0.25	**0.88**	**0.00**	**0.89**	**0.00**
Benzaldehyde	-0.44	0.23	-0.28	0.47	0.57	0.11	0.16	0.69	**-0.75**	**0.02**	0.15	0.70	-0.15	0.70	-0.15	0.71	0.44	0.24
5-Hydroxymethylfurfural	0.43	0.25	0.21	0.59	-0.31	0.41	**0.97**	**0.00**	-0.12	0.75	**0.92**	**0.00**	0.61	0.08	**0.91**	**0.00**	0.62	0.07
Furfural	0.54	0.14	0.43	0.25	-0.39	0.29	**0.98**	**0.00**	-0.13	0.73	**0.93**	**0.00**	0.40	0.29	**0.89**	**0.00**	**0.89**	**0.00**
Ethyl iso-allocholate	0.40	0.28	0.40	0.29	-0.56	0.12	-0.49	0.18	**0.82**	**0.01**	-0.44	0.23	-0.26	0.51	-0.16	0.69	-0.44	0.23
Ethyl Acetate	**0.82**	**0.01**	**0.77**	**0.02**	**-0.84**	**0.00**	0.44	0.24	0.57	0.11	0.49	0.18	-0.06	0.87	0.66	0.05	0.52	0.15
Ethyl caproate	**-0.75**	**0.02**	-0.67	0.05	**0.71**	**0.03**	-0.46	0.21	-0.39	0.31	-0.40	0.29	-0.28	0.47	-0.57	0.11	-0.43	0.25
Ethyl lactate	**-0.75**	**0.02**	-0.58	0.10	0.66	0.05	-0.40	0.28	-0.46	0.21	-0.26	0.50	-0.47	0.20	-0.47	0.20	-0.32	0.40
Ethyl 9-hexadecenoate	**0.87**	**0.00**	**0.76**	**0.02**	**-0.90**	**0.00**	0.12	0.77	**0.84**	**0.00**	0.04	0.91	0.19	0.62	0.36	0.33	0.10	0.80

Cyclopropanetetradecanoic acid, 2-octyl-,methyl ester	**-0.96**	**0.00**	**-0.84**	**0.00**	**0.95**	**0.00**	-0.49	0.18	-0.68	0.05	-0.44	0.24	-0.26	0.51	**-0.70**	**0.04**	-0.44	0.23
[1,1'-Bicyclopropyl]-2-octanoic acid, 2'-hexyl-, methyl ester	0.36	0.34	0.44	0.23	-0.58	0.10	-0.47	0.20	**0.83**	**0.01**	-0.43	0.25	-0.25	0.52	-0.15	0.70	-0.43	0.25
9-Octadecenoic acid, (2-phenyl-1,3-dioxolan-4-yl) methyl ester, trans	0.40	0.28	0.39	0.30	-0.56	0.12	-0.49	0.18	**0.83**	**0.01**	-0.44	0.24	-0.26	0.51	-0.16	0.69	-0.44	0.23
Ethyl benzeneacetate	-0.47	0.20	-0.45	0.23	0.42	0.26	-0.16	0.68	-0.39	0.29	-0.01	0.97	-0.31	0.42	-0.16	0.68	-0.23	0.55
β -Phenethyl acetate	0.23	0.55	0.44	0.23	-0.32	0.40	0.41	0.27	0.04	0.92	0.48	0.20	-0.18	0.65	0.39	0.30	0.61	0.08
Ethyl dodecanoate	-0.40	0.28	-0.51	0.16	0.52	0.15	-0.49	0.18	-0.28	0.46	-0.48	0.19	-0.20	0.61	-0.54	0.13	-0.42	0.26
Ethyl tetradecanoate	-0.37	0.33	-0.26	0.49	0.28	0.47	-0.06	0.88	-0.26	0.50	0.10	0.81	-0.37	0.33	-0.07	0.86	-0.03	0.94
Ethyl hexadecanoate	0.55	0.13	0.24	0.53	-0.45	0.22	0.46	0.21	0.33	0.39	0.47	0.20	0.23	0.54	0.58	0.10	0.28	0.47
DL-Phenylalanine, N-chlorodifluoroacetyl-, ethyl ester	**0.86**	**0.00**	**0.85**	**0.00**	**-0.79**	**0.01**	0.66	0.05	0.32	0.41	0.64	0.06	0.10	0.79	**0.77**	**0.02**	**0.79**	**0.01**
9-Octadecenoic acid, (2-phenyl-1,3-	-0.45	0.23	-0.46	0.22	0.51	0.16	-0.28	0.47	-0.29	0.46	-0.39	0.30	0.16	0.69	-0.49	0.18	-0.27	0.48

dioxolan-4-yl) methyl ester, cis																		
9-Octadecenoic acid, (2-phenyl-1,3-dioxolan-4-yl) methyl ester, trans	-0.58	0.10	-0.54	0.14	**0.69**	**0.04**	0.29	0.45	**-0.83**	**0.01**	0.31	0.42	0.05	0.89	-0.01	0.99	0.27	0.48
Ethyl succinate	**-0.85**	**0.00**	**-0.68**	**0.04**	**0.86**	**0.00**	-0.44	0.24	-0.67	0.05	-0.34	0.37	-0.47	0.20	-0.62	0.07	-0.24	0.54
Ethyl (9E)-9-octadecenoate	-0.38	0.32	-0.29	0.46	0.38	0.31	-0.13	0.73	-0.39	0.30	-0.07	0.86	-0.27	0.49	-0.24	0.54	0.00	1.00
Ethyl 9,12,15-octadecatrienoate	-0.36	0.35	-0.28	0.46	0.29	0.44	-0.13	0.74	-0.22	0.57	-0.04	0.92	-0.26	0.51	-0.17	0.65	-0.09	0.82
9,12,15-Octadecatrienoic acid, 2,3-dihydroxypropyl ester, (Z,Z,Z)-	**-0.91**	**0.00**	**-0.80**	**0.01**	**0.91**	**0.00**	-0.46	0.22	-0.65	0.06	-0.41	0.27	-0.24	0.53	-0.66	0.06	-0.42	0.27
Ethyl-9,12-octadecadienoate	-0.39	0.30	-0.30	0.44	0.48	0.19	-0.14	0.72	-0.52	0.15	-0.29	0.45	0.29	0.45	-0.36	0.35	-0.14	0.71
Dibutyl phthalate	**-0.92**	**0.00**	**-0.81**	**0.01**	**0.92**	**0.00**	-0.46	0.21	-0.66	0.05	-0.42	0.26	-0.24	0.53	-0.66	0.05	-0.42	0.26
Phthalic acid, di (2-propylpentyl) ester	**-0.87**	**0.00**	**-0.73**	**0.02**	**0.76**	**0.02**	**-0.81**	**0.01**	-0.27	0.48	**-0.70**	**0.03**	-0.50	0.17	**-0.86**	**0.00**	**-0.72**	**0.03**
[1,1'-Bicyclopropyl]-2-octanoic acid, 2'-hexyl-, methyl ester	0.33	0.38	0.46	0.21	-0.58	0.10	-0.45	0.22	**0.84**	**0.00**	-0.41	0.28	-0.24	0.54	-0.14	0.71	-0.41	0.27

Ethyl 13-methyl-tetradecanoate	-0.57	0.11	-0.45	0.22	0.43	0.25	**-0.87**	**0.00**	0.05	0.90	**-0.78**	**0.01**	-0.46	0.22	**-0.79**	**0.01**	**-0.79**	**0.01**
n-Propyl 9,12-octadecadienoate	-0.34	0.37	-0.46	0.21	0.37	0.33	0.01	0.98	-0.30	0.43	0.06	0.88	0.05	0.90	-0.03	0.93	-0.19	0.62
Butyl 9,12-octadecadienoate	**-0.89**	**0.00**	**-0.79**	**0.01**	**0.89**	**0.00**	-0.44	0.23	-0.64	0.06	-0.40	0.28	-0.23	0.55	-0.64	0.06	-0.40	0.28
Ethyl 9.cis.,11.trans.-octadecadienoate	**0.85**	**0.00**	0.62	0.07	**-0.72**	**0.03**	0.35	0.36	0.42	0.26	0.36	0.34	0.03	0.95	0.57	0.11	0.39	0.30
Nitrosomethane	**-0.74**	**0.02**	-0.62	0.07	**0.71**	**0.03**	-0.39	0.30	-0.52	0.15	-0.35	0.35	-0.21	0.60	-0.56	0.11	-0.36	0.34
Benzeneethanamine, 3-fluoro-ά,5-dihydroxy-N-methyl	0.40	0.29	0.40	0.28	-0.55	0.12	-0.49	0.18	**0.79**	**0.01**	-0.44	0.24	-0.25	0.51	-0.15	0.69	-0.44	0.23
(2-Aziridinylethyl) amine	**0.74**	**0.02**	0.62	0.07	**-0.81**	**0.01**	0.47	0.20	0.63	0.07	0.59	0.09	-0.13	0.75	**0.74**	**0.02**	0.42	0.26
Phenol	0.40	0.29	0.38	0.31	-0.53	0.14	-0.48	0.19	0.75	0.02	-0.44	0.24	-0.25	0.51	-0.15	0.70	-0.44	0.24
(2S,2'S)-2,2'-Bis [1,4,7,10,13-pentaoxacycl-opentadecane]	**-0.92**	**0.00**	**-0.81**	**0.01**	**0.92**	**0.00**	-0.47	0.20	-0.66	0.05	-0.43	0.25	-0.25	0.52	-0.68	0.05	-0.43	0.25

r, Pearson correlation coefficient; significant correlations ($P < 0.05$) are in bold.

Table S2 (continued) Spearman’s correlation coefficients between shared microorganisms and volatile flavors compounds.

Microorganisms	*Staphylococcus*		*Thermoactinomyces*		*Virgibacillus*		*Pseudogracilibacillus*		*unclassified_f__ Bacillaceae*		*Aspergillus*		*Byssochlamys*		*Issatchenkia*	
Volatile compounds	*r*	*p*	*r*	*p*	*r*	*p*	*r*	*p*	*r*	*p*	*r*	*p*	*r*	*p*	*r*	*p*
Hexadecanoic acid	0.48	0.19	0.07	0.85	0.33	0.39	-0.46	0.21	-0.19	0.62	0.59	0.09	0.25	0.52	-0.46	0.21
Acetic acid	**-0.73**	**0.03**	**-0.68**	**0.04**	**-0.81**	**0.01**	-0.35	0.35	-0.42	0.26	0.58	0.10	-0.52	0.15	**-0.74**	**0.02**
Isobutyric acid	**-0.83**	**0.01**	**-0.77**	**0.01**	**-0.92**	**0.00**	-0.40	0.28	-0.48	0.19	0.41	0.28	-0.61	0.08	-0.64	0.06
Butyric acid	**-0.82**	**0.01**	**-0.77**	**0.02**	**-0.91**	**0.00**	-0.40	0.29	-0.48	0.19	0.40	0.28	-0.61	0.08	-0.63	0.07
Hexanoic acid	-0.41	0.28	-0.31	0.41	-0.39	0.30	-0.21	0.59	-0.16	0.68	0.50	0.17	-0.18	0.64	-0.61	0.08
1-Pentanol	0.07	0.85	**0.84**	**0.00**	0.57	0.11	**0.83**	**0.01**	**0.96**	**0.00**	-0.29	0.45	0.62	0.08	0.61	0.08
Phenylethyl Alcohol	0.15	0.70	-0.09	0.82	0.06	0.87	-0.14	0.71	-0.16	0.68	**0.72**	**0.03**	0.10	0.80	-0.57	0.11
Ethanol	-0.63	0.07	-0.59	0.10	**-0.69**	**0.04**	-0.30	0.43	-0.37	0.33	0.31	0.42	-0.45	0.22	-0.43	0.24
1-Heptanol	**0.80**	**0.01**	-0.02	0.95	0.33	0.39	-0.38	0.31	-0.46	0.22	0.14	0.72	0.00	0.99	-0.11	0.78

Nicotinyl alcohol	**0.81**	**0.01**	-0.05	0.90	0.36	0.34	-0.39	0.30	-0.47	0.20	-0.01	0.97	0.04	0.92	-0.04	0.93
3-Furanmethanol	**-0.72**	**0.03**	0.17	0.66	-0.18	0.65	0.47	0.20	0.58	0.10	0.03	0.93	0.12	0.76	0.03	0.94
Undecaethylene glycol	**0.80**	**0.01**	-0.06	0.88	0.34	0.37	-0.40	0.28	-0.48	0.19	-0.09	0.81	0.01	0.98	0.03	0.94
Benzaldehyde	-0.14	0.72	**-0.74**	**0.02**	-0.60	0.09	-0.68	0.05	**-0.81**	**0.01**	0.65	0.06	-0.56	0.12	**-0.80**	**0.01**
5-Hydroxymethylfurfural	0.67	0.05	-0.09	0.82	0.28	0.46	-0.37	0.33	-0.44	0.24	-0.39	0.30	-0.02	0.96	0.23	0.56
Furfural	**0.80**	**0.01**	-0.06	0.88	0.35	0.36	-0.40	0.29	-0.48	0.19	-0.09	0.82	0.02	0.96	0.02	0.95
Ethyl iso-allocholate	0.04	0.92	**0.84**	**0.01**	0.60	0.09	**0.81**	**0.01**	**0.97**	**0.00**	-0.30	0.43	0.64	0.06	0.61	0.08
Ethyl Acetate	**0.79**	**0.01**	0.67	0.05	**0.85**	**0.00**	0.32	0.39	0.39	0.30	-0.20	0.60	0.61	0.08	0.46	0.22
Ethyl caproate	-0.68	0.04	-0.49	0.18	-0.60	0.09	-0.27	0.48	-0.22	0.57	0.13	0.74	-0.32	0.40	-0.32	0.41
Ethyl lactate	-0.55	0.12	-0.49	0.18	-0.61	0.08	-0.28	0.46	-0.28	0.47	0.52	0.15	-0.28	0.47	-0.61	0.08
Ethyl 9-hexadecenoate	0.55	0.12	**0.89**	**0.00**	**0.88**	**0.00**	0.61	0.08	**0.75**	**0.02**	-0.44	0.24	0.67	0.05	**0.75**	**0.02**

Cyclopropanetetradecanoic acid, 2-octyl-,methyl ester	**-0.83**	**0.01**	**-0.78**	**0.01**	**-0.92**	**0.00**	-0.40	0.28	-0.48	0.19	0.41	0.28	-0.62	0.08	-0.67	0.05
[1,1'-Bicyclopropyl]-2-octanoic acid, 2'-hexyl-, methyl ester	0.11	0.79	**0.89**	**0.00**	0.61	0.08	**0.69**	**0.04**	**0.99**	**0.00**	-0.23	0.54	**0.73**	**0.02**	0.52	0.15
9-Octadecenoic acid, (2-phenyl-1,3-dioxolan-4-yl) methyl ester, trans	0.02	0.95	**0.83**	**0.01**	0.61	0.08	**0.80**	**0.01**	**0.97**	**0.00**	-0.31	0.42	0.64	0.06	0.60	0.08
Ethyl benzeneacetate	-0.34	0.38	-0.40	0.28	-0.44	0.23	-0.06	0.87	-0.27	0.49	0.47	0.21	-0.28	0.46	-0.48	0.19
β -Phenethyl acetate	0.51	0.16	0.08	0.83	0.27	0.49	-0.41	0.27	-0.18	0.63	0.64	0.06	0.31	0.42	-0.53	0.14
Ethyl dodecanoate	-0.65	0.06	-0.39	0.30	-0.48	0.19	0.26	0.49	-0.09	0.82	-0.15	0.71	-0.52	0.15	0.03	0.94
Ethyl tetradecanoate	-0.21	0.59	-0.34	0.36	-0.29	0.45	-0.24	0.53	-0.27	0.48	0.54	0.13	-0.14	0.73	-0.64	0.06
Ethyl hexadecanoate	0.34	0.37	0.17	0.66	0.51	0.16	0.20	0.61	0.07	0.85	-0.60	0.09	0.15	0.71	0.53	0.14
DL-Phenylalanine, N-chlorodifluoroacetyl-, ethyl ester	**0.97**	**0.00**	0.52	0.15	**0.72**	**0.03**	0.14	0.73	0.11	0.78	-0.05	0.89	0.41	0.27	0.28	0.47
9-Octadecenoic acid, (2-phenyl-1,3-	-0.57	0.11	-0.45	0.22	-0.48	0.19	-0.29	0.46	-0.25	0.51	0.15	0.71	-0.36	0.34	-0.22	0.57

dioxolan-4-yl) methyl ester, cis																
9-Octadecenoic acid, (2-phenyl-1,3-dioxolan-4-yl) methyl ester, trans	-0.22	0.57	**-0.88**	**0.00**	**-0.69**	**0.04**	**-0.76**	**0.02**	**-0.92**	**0.00**	0.36	0.34	-0.65	0.06	**-0.71**	**0.03**
Ethyl succinate	**-0.70**	**0.04**	**-0.74**	**0.02**	**-0.82**	**0.01**	-0.37	0.33	-0.51	0.17	0.56	0.12	-0.58	0.10	**-0.77**	**0.01**
Ethyl (9E)-9-octadecenoate	-0.29	0.45	-0.43	0.25	-0.41	0.27	-0.24	0.54	-0.35	0.36	0.66	0.05	-0.28	0.47	-0.60	0.09
Ethyl 9,12,15-octadecatrienoate	-0.28	0.47	-0.31	0.41	-0.28	0.47	-0.24	0.54	-0.21	0.59	0.51	0.16	-0.10	0.80	-0.48	0.19
9,12,15-Octadecatrienoic acid, 2,3-dihydroxypropyl ester, (Z,Z,Z)-	**-0.78**	**0.01**	**-0.73**	**0.03**	**-0.86**	**0.00**	-0.38	0.32	-0.45	0.22	0.36	0.34	-0.57	0.11	-0.57	0.11
Ethyl-9,12-octadecadienoate	-0.20	0.60	-0.29	0.45	-0.57	0.11	-0.19	0.62	-0.29	0.45	0.08	0.84	-0.44	0.24	-0.11	0.78
Dibutyl phthalate	**-0.79**	**0.01**	**-0.74**	**0.02**	**-0.87**	**0.00**	-0.38	0.31	-0.46	0.21	0.37	0.33	-0.58	0.10	-0.58	0.10
Phthalic acid, di (2-propylpentyl) ester	**-0.95**	**0.00**	-0.45	0.23	-0.67	0.05	-0.08	0.84	-0.02	0.95	0.35	0.36	-0.30	0.44	-0.48	0.19
[1,1'-Bicyclopropyl]-2-octanoic acid, 2'-hexyl-, methyl ester	0.13	0.73	**0.90**	**0.00**	0.61	0.08	0.60	0.09	**0.98**	**0.00**	-0.19	0.62	**0.78**	**0.01**	0.46	0.21

Ethyl 13-methyl-tetradecanoate	**-0.76**	**0.02**	-0.04	0.91	-0.38	0.31	0.29	0.45	0.35	0.36	0.14	0.73	-0.07	0.86	-0.16	0.68
n-Propyl 9,12-octadecadienoate	-0.29	0.45	-0.37	0.32	-0.31	0.41	-0.14	0.72	-0.26	0.49	-0.02	0.95	-0.26	0.50	-0.05	0.90
Butyl 9,12-octadecadienoate	**-0.76**	**0.02**	**-0.71**	**0.03**	**-0.84**	**0.00**	-0.37	0.33	-0.44	0.23	0.31	0.41	-0.56	0.12	-0.53	0.15
Ethyl 9.cis.,11.trans.-octadecadienoate	0.62	0.07	0.53	0.14	**0.69**	**0.04**	0.66	0.05	0.34	0.37	-0.38	0.32	0.27	0.48	0.66	0.05
Nitrosomethane	-0.67	0.05	-0.63	0.07	**-0.74**	**0.02**	-0.33	0.39	-0.39	0.30	0.61	0.08	-0.47	0.20	**-0.73**	**0.03**
Benzeneethanamine, 3-fluoro-á,5-dihydroxy-N-methyl	0.06	0.87	**0.84**	**0.00**	0.58	0.10	**0.82**	**0.01**	**0.97**	**0.00**	-0.29	0.44	0.62	0.07	0.61	0.08
(2-Aziridinylethyl) amine	**0.68**	**0.04**	0.59	0.10	**0.88**	**0.00**	0.26	0.50	0.38	0.32	-0.34	0.37	0.65	0.06	0.45	0.23
Phenol	0.05	0.89	**0.81**	**0.01**	0.55	0.13	**0.87**	**0.00**	**0.94**	**0.00**	-0.31	0.42	0.57	0.11	0.63	0.07
(2S,2'S)-2,2'-Bis [1,4,7,10,13-pentaoxacycl-opentadecane]	**-0.80**	**0.01**	**-0.75**	**0.02**	**-0.89**	**0.00**	-0.39	0.30	-0.47	0.20	0.47	0.21	-0.58	0.10	-0.66	0.05

r, Pearson correlation coefficient; significant correlations ($P < 0.05$) are in bold.

Table S2 (continued) Spearman's correlation coefficients between shared microorganisms and volatile flavors compounds.

Microorganisms	*Monascus*		*Pichia*		*Thermoascus*		*Thermomyces*		*Nigrospora*		*Rasamsonia*	
Volatile compounds	*r*	*p*	*r*	*p*	*r*	*p*	*r*	*p*	*r*	*p*	*r*	*p*
Hexadecanoic acid	**0.68**	**0.04**	-0.12	0.75	-0.17	0.65	0.67	0.05	0.17	0.67	0.12	0.77
Acetic acid	-0.42	0.27	-0.55	0.12	0.48	0.20	0.54	0.14	**0.83**	**0.01**	**0.89**	**0.00**
Isobutyric acid	-0.47	0.20	-0.63	0.07	**0.71**	**0.03**	0.40	0.28	0.50	0.17	0.59	0.10
Butyric acid	-0.47	0.20	-0.62	0.07	**0.69**	**0.04**	0.40	0.28	0.49	0.18	0.58	0.10
Hexanoic acid	-0.28	0.47	-0.44	0.24	0.35	0.35	0.34	0.37	0.64	0.06	**0.68**	**0.04**
1-Pentanol	-0.47	0.20	0.22	0.57	-0.54	0.13	**-0.71**	**0.03**	-0.25	0.52	-0.29	0.44
Phenylethyl Alcohol	0.18	0.64	-0.41	0.28	-0.19	0.62	**0.73**	**0.03**	0.57	0.11	0.53	0.14
Ethanol	-0.36	0.34	-0.48	0.19	0.39	0.30	0.37	0.33	0.38	0.32	0.43	0.25
1-Heptanol	**0.98**	**0.00**	0.26	0.51	-0.11	0.77	0.47	0.20	-0.24	0.54	-0.28	0.47

Nicotinyl alcohol	**0.92**	**0.00**	0.26	0.50	-0.15	0.70	0.38	0.32	-0.24	0.53	-0.29	0.45
3-Furanmethanol	**-0.91**	**0.00**	-0.32	0.40	0.14	0.72	-0.41	0.27	0.18	0.64	0.22	0.57
Undecaethylene glycol	**0.93**	**0.00**	0.35	0.35	-0.18	0.64	0.33	0.39	-0.25	0.52	-0.29	0.44
Benzaldehyde	0.35	0.36	-0.51	0.16	0.62	0.07	**0.87**	**0.00**	0.29	0.44	0.34	0.37
5-Hydroxymethylfurfural	**0.74**	**0.02**	0.49	0.18	-0.26	0.50	0.08	0.84	-0.23	0.56	-0.27	0.48
Furfural	**0.92**	**0.00**	0.33	0.38	-0.18	0.65	0.33	0.39	-0.25	0.52	-0.29	0.44
Ethyl iso-allocholate	-0.48	0.20	0.22	0.56	-0.56	0.12	**-0.71**	**0.03**	-0.25	0.52	-0.30	0.44
Ethyl Acetate	0.45	0.23	0.34	0.36	-0.67	0.05	-0.16	0.68	-0.43	0.25	-0.52	0.15
Ethyl caproate	-0.47	0.20	-0.53	0.14	0.52	0.16	0.13	0.74	0.10	0.80	0.17	0.66
Ethyl lactate	-0.43	0.25	-0.69	0.04	0.32	0.39	0.48	0.19	0.59	0.09	0.63	0.07
Ethyl 9-hexadecenoate	0.15	0.70	0.62	0.07	**-0.78**	**0.01**	-0.59	0.09	-0.45	0.22	-0.54	0.13

Cyclopropanetetradecanoic acid, 2-octyl-,methyl ester	-0.47	0.20	-0.63	0.07	**0.77**	**0.02**	0.38	0.31	0.50	0.17	0.59	0.09
[1,1'-Bicyclopropyl]-2-octanoic acid, 2'-hexyl-, methyl ester	-0.46	0.21	0.11	0.77	-0.51	0.16	**-0.69**	**0.04**	-0.24	0.53	-0.29	0.45
9-Octadecenoic acid, (2-phenyl-1,3-dioxolan-4-yl) methyl ester, trans	-0.47	0.20	0.22	0.56	-0.56	0.12	**-0.71**	**0.03**	-0.25	0.52	-0.30	0.44
Ethyl benzeneacetate	-0.30	0.43	-0.34	0.37	-0.05	0.89	0.49	0.18	**0.88**	**0.00**	**0.87**	**0.00**
β -Phenethyl acetate	0.50	0.18	-0.30	0.43	-0.26	0.51	0.70	0.04	0.42	0.26	0.36	0.34
Ethyl dodecanoate	-0.44	0.24	-0.04	0.91	0.55	0.12	-0.22	0.57	-0.11	0.78	-0.03	0.94
Ethyl tetradecanoate	-0.14	0.72	-0.46	0.22	0.16	0.69	0.52	0.15	**0.75**	**0.02**	**0.76**	**0.02**
Ethyl hexadecanoate	0.32	0.41	0.56	0.12	-0.28	0.47	-0.37	0.32	-0.47	0.20	-0.50	0.17
DL-Phenylalanine, N-chlorodifluoroacetyl-, ethyl ester	**0.74**	**0.02**	0.38	0.32	-0.46	0.21	0.04	0.92	-0.42	0.26	-0.50	0.17
9-Octadecenoic acid, (2-phenyl-1,3-	-0.19	0.62	-0.13	0.73	0.15	0.70	0.25	0.52	0.26	0.50	0.29	0.45

dioxolan-4-yl) methyl ester, cis												
9-Octadecenoic acid, (2-phenyl-1,3-dioxolan-4-yl) methyl ester, trans	0.26	0.50	-0.40	0.28	**0.71**	**0.03**	0.67	0.05	0.33	0.39	0.40	0.29
Ethyl succinate	-0.35	0.35	**-0.73**	**0.03**	**0.83**	**0.01**	0.51	0.16	0.46	0.21	0.55	0.12
Ethyl (9E)-9-octadecenoate	-0.08	0.84	-0.38	0.31	-0.07	0.87	**0.73**	**0.02**	**0.81**	**0.01**	**0.80**	**0.01**
Ethyl 9,12,15-octadecatrienoate	-0.16	0.69	-0.38	0.32	-0.17	0.66	0.58	0.10	**0.72**	**0.03**	**0.70**	**0.04**
9,12,15-Octadecatrienoic acid, 2,3-dihydroxypropyl ester, (Z,Z,Z)-	-0.44	0.23	-0.59	0.09	0.63	0.07	0.38	0.31	0.43	0.25	0.51	0.16
Ethyl-9,12-octadecadienoate	-0.04	0.92	-0.07	0.86	0.37	0.33	0.09	0.82	-0.03	0.94	0.02	0.97
Dibutyl phthalate	-0.45	0.22	-0.60	0.09	0.64	0.06	0.39	0.30	0.44	0.23	0.52	0.15
Phthalic acid, di (2-propylpentyl) ester	**-0.79**	**0.01**	-0.66	0.05	0.51	0.16	0.13	0.74	0.48	0.19	0.55	0.12
[1,1'-Bicyclopropyl]-2-octanoic acid, 2'-hexyl-, methyl ester	-0.44	0.24	0.04	0.91	-0.48	0.20	-0.65	0.06	-0.23	0.55	-0.27	0.48

Ethyl 13-methyl-tetradecanoate	**-0.85**	**0.00**	-0.42	0.27	0.37	0.32	-0.27	0.49	0.27	0.49	0.33	0.39
n-Propyl 9,12-octadecadienoate	-0.16	0.68	-0.05	0.90	-0.17	0.67	0.21	0.58	0.34	0.38	0.32	0.40
Butyl 9,12-octadecadienoate	-0.43	0.25	-0.58	0.10	0.63	0.07	0.35	0.36	0.36	0.35	0.44	0.24
Ethyl 9.cis.,11.trans.-octadecadienoate	0.36	0.35	0.66	0.06	-0.65	0.06	-0.31	0.42	-0.42	0.26	-0.50	0.17
Nitrosomethane	-0.38	0.31	-0.51	0.16	0.38	0.31	0.55	0.12	**0.88**	**0.00**	**0.93**	**0.00**
Benzeneethanamine, 3-fluoro-ά,5-dihydroxy-N-methyl	-0.47	0.20	0.22	0.57	-0.55	0.13	**-0.71**	**0.03**	-0.25	0.52	-0.30	0.44
(2-Aziridinylethyl) amine	0.34	0.37	0.30	0.44	-0.59	0.10	-0.27	0.49	-0.41	0.27	-0.49	0.18
Phenol	-0.47	0.20	0.26	0.50	-0.55	0.12	**-0.71**	**0.03**	-0.25	0.52	-0.29	0.44
(2S,2'S)-2,2'-Bis [1,4,7,10,13-pentaoxacycl-opentadecane]	-0.46	0.21	-0.61	0.08	0.59	0.09	0.47	0.20	0.61	0.08	0.68	0.04

r, Pearson correlation coefficient; significant correlations ($P < 0.05$) are in bold.

Supplementary Figure

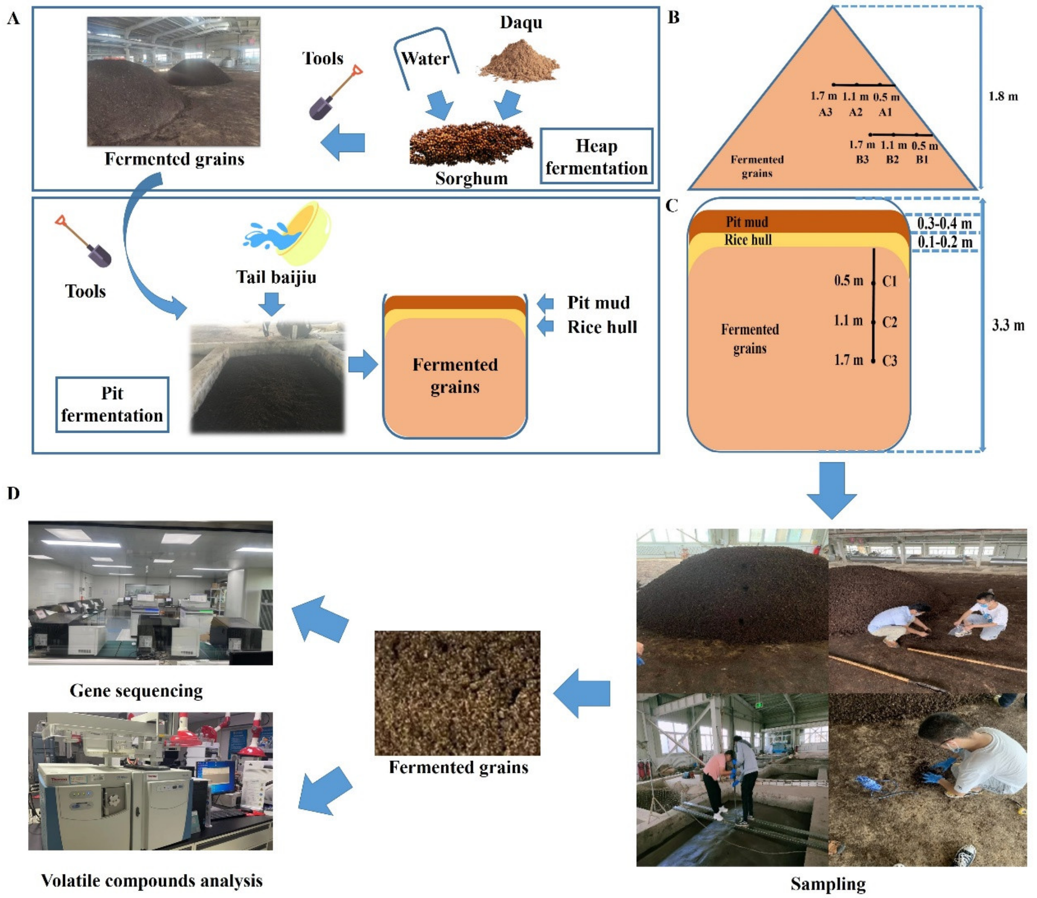

Figure S1 Schema of Sauce-flavor Baijiu production (A). Sampling positions during heap fermentation (B) and pit fermentation (C). Sampling and experimental processes (D). The prefix "A" and "B" indicated that samples were collected from the upper and lower layers in heap fermentation respectively. At each location, three paralleled fermented grains samples were collected, and all these samples were grinded and mixed as fermented grains-H sample. The prefix "C" indicated that samples were collected from the upper and middle layers in pit fermentation. The triplicate fermented grains samples in pit fermentation were collected at 0 and 30 days, and then grinded and mixed as fermented grains-P0 and fermented grains-P30 samples, respectively.

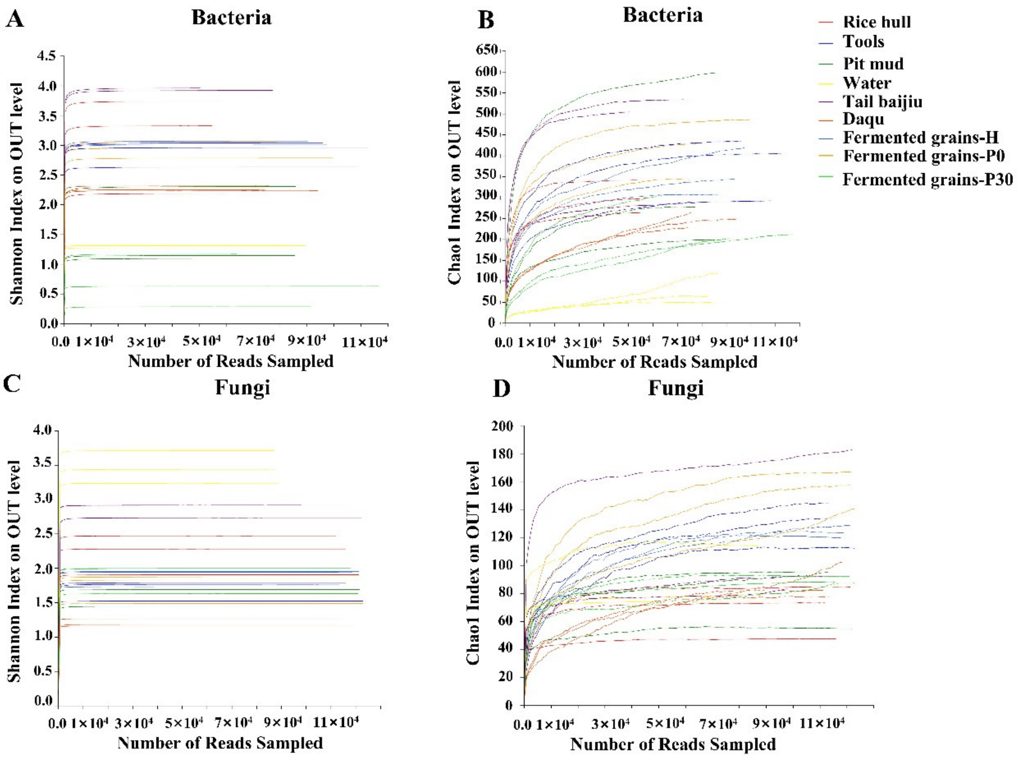

Figure S2 Rarefaction curves of Shannon index (A, C) and Chao1 index (B, D) for different samples.
